# Chemical EOR with Methyl Ester Sulfonate: Achieving Residual Oil Saturation via 2–4-Order Capillary Number Increase

**DOI:** 10.1021/acsphyschemau.5c00087

**Published:** 2025-12-17

**Authors:** Farizal Hakiki, Muhamad Raihan Al Fikri, Veni Dwi Amelia Putri, Indra Gunawan, Witta Kartika Restu, Muslim Abdurrahman

**Affiliations:** † 34914 National Yang Ming Chiao Tung University (NYCU), Disaster Prevention & Water Environment Research Center (DPWE), Hsinchu 300, Taiwan; ‡ National Yang Ming Chiao Tung University (NYCU), Civil Engineering Department, Hsinchu 300, Taiwan; § Department of Petroleum Engineering, 175474 Universitas Islam Riau, Engineering Faculty, Pekanbaru, Riau 28284, Indonesia; ∥ National Research and Innovation Agency (BRIN), Research Center for Chemistry, Serpong, South Tangerang 15314, Indonesia

**Keywords:** chemical enhanced oil recovery, methyl ester sulfonate, spontaneous imbibition, high salinity, capillary number

## Abstract

This study extends the application of methyl ester sulfonate (MES), a biodegradable, anionic surfactant derived from renewable resources, for chemically enhanced oil recovery (EOR) in high-salinity (up to 700 mM NaCl) and high-temperature (80 °C) conditions. MES is demonstrated to reduce oil–water interfacial tension to low values (∼0.02 mN/m at 80 °C), alter sandstone wettability to preferentially water-wet, and form stable Winsor III microemulsions. We highlight a key advancement by systematically compiling and comparing published capillary desaturation data, showing that increasing the capillary number by a factor of 10^2^–10^4^ enables the system to approach irreducible water or residual oil saturation. Our experimental results align with this trend, where MES significantly increases capillary numbers through synergistic effects of interfacial tension reduction and wettability alteration. Zeta potential measurements confirm colloidal stability across a broad concentration and salinity range, while thermal analysis supports MES stability up to 90 °C. Spontaneous imbibition tests show oil recovery exceeding 28% under optimized salinity, further validating the MES efficacy. This work not only expands the operational envelope of MES for chemical EOR but also reinforces the mechanistic link between the capillary number scaling and residual oil displacement. By integrating lab-scale results with a broad data set from the literature, this study delivers a compelling foundation for deploying MES in field-scale EOR operations, particularly in mature reservoirs where sustainable and cost-effective solutions are critical.

## Graphical abstract

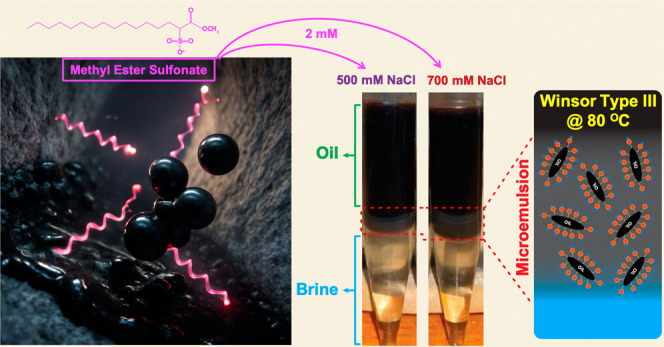

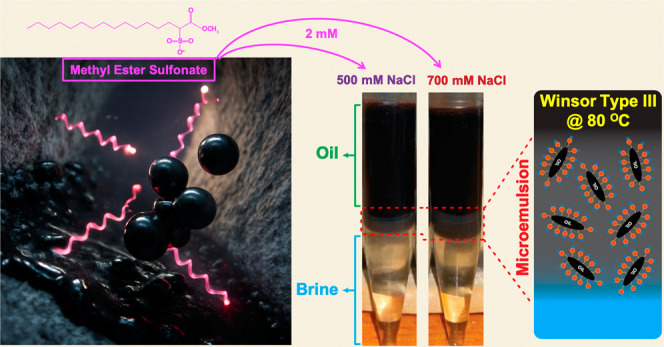

## 1 Introduction

Enhanced oil recovery (EOR) employs advanced techniques such as thermal, gas, and chemical injections to modify the physical and chemical properties of the oil-rock-water system and their interactions, thereby promoting favorable oil recovery.^1–3^ Chemical EOR specifically targets the reduction of interfacial tension σ and the alteration of wettability toward more water-wet conditions, as indicated by changes in the contact angle θ. These modifications lead to increases in the trapping number *N*_T_ and capillary number *N*_Ca_, resulting in a subsequent reduction in residual oil saturation^2,4,5^ (Figure 1a). Wettability alterations from preferentially oil-wet to preferentially water-wet conditions can also be observed through the evolution of relative permeabilities (Figure 1b–e). The capillary number *N*_Ca_ correspondingly increases during these relative permeability evolutions.

**Figure 1:**
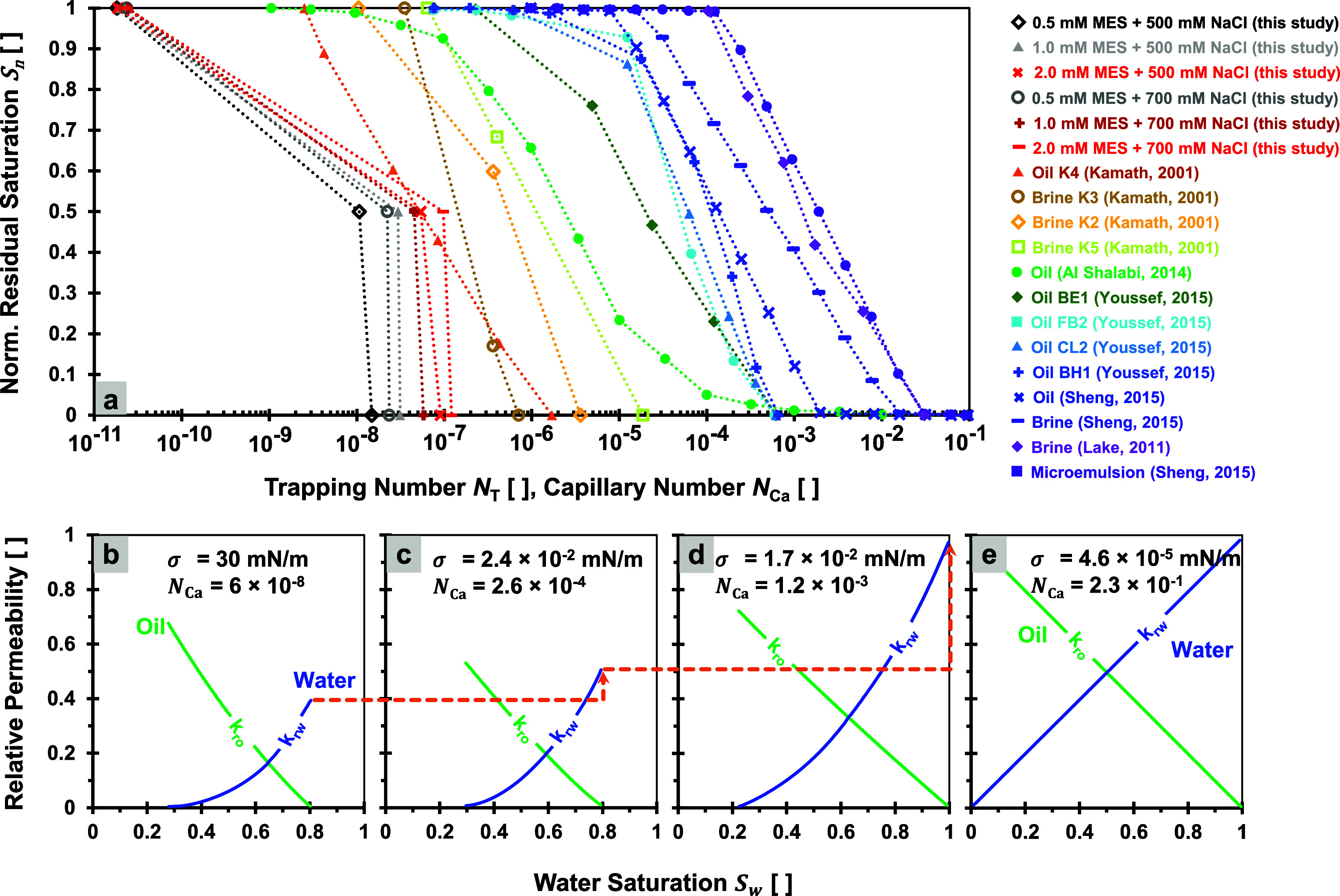
Capillary desaturation curve and relative permeability evolutions. (a) Effect of increasing trapping and capillary number on residual oil saturation. Data sources: refs 5,86–90 . (b–e) Evolution of relative permeability when the capillary number increases. Data source: ref 5. Normalized residual oil saturation *S*_*n*_ is computed with eq 8.

The capillary number *N*_Ca_ is closely related to the trapping number *N*_T_, reducing to *N*_T_ = *N*_Ca_ in the absence of a buoyancy contribution, which is quantified by the Bond number *N*_Bo_. The general formulations of these dimensionless numbers are as follows:^2,5–9^

$$N_{T}=N_{\mathrm{Ca}}+N_{\mathrm{Bo}}\sin\;\alpha$$ 1

NCa=FvFc=μinvvinvσowcosθow,NCa=μinvvinvσow,NCa=μinvvinvσow(1+cosθow) 2

$$N_{\mathrm{Bo}}=\frac{F_{b}}{F_{c}}=\frac{(\rho_{w}-\rho_{o})r^{2}}{\sigma_{\mathrm{ow}}\cos\;\theta_{\mathrm{ow}}}$$ 3

where these numbers represent the dimensionless ratios of viscous, buoyancy, and capillary forces. In fact, there are also dozens of capillary number *N*_Ca_ definitions.^9–11^ The latter corresponds to our proposed capillary number and is discussed further in the Results and Discussion section.

The capillary number *N*_Ca_ expresses the ratio between the viscous force *F*_v_ and capillary force *F*_c_. Oil recovery typically increases (i.e., residual oil saturation decreases) when *N*_Ca_ is enhanced. This can be achieved by increasing the viscosity of the invading fluid μ_inv_ (e.g., through polymer injection), by increasing the invading-phase velocity *v*_inv_ (e.g., waterflooding or surfactant–polymer flooding), by reducing the oil–water interfacial tension σ_ow_ (e.g., surfactant flooding), or by altering the rock wettability, which affects the internal oil-in-water contact angle θ_ow_.

The approximate form of the capillary number in eq 2, where cos θ is often omitted, is referred to as the *microscopic capillary number*. This form is applicable under slow fluid-displacement conditions, where the invading-phase velocity *v*_inv_ is treated as the interstitial (pore-scale) velocity rather than the Darcy velocity.^12^ However, the cosine factor must be included if there are significant wettability alterations. The contact angle θ is an oil droplet measured under a water environment.^13^ This setup is more delicate compared to that of the water droplet in air.

In contact angle measurements, a buoyancy number *N*_Bo_ less than 1 suggests that surface tension-induced forces *F*_c_ dominate over buoyancy forces *F*_b_. Therefore, a small droplet size *r* of oil or water is expected. As a result, the droplet shape is mainly governed by surface tension and its interaction with the solid surface rather than by buoyancy effects. In a bigger scale such as fluid flow in porous media, buoyancy effects would take a serious effect if there is a certain inclination angle α due to a significant density difference between water and oil (ρ_w_ – ρ_o_). This may be found in a vertical flow (α = 90°) such that a gravity drainage mechanism prevails.

Surfactant flooding, one of the methods in chemical EOR, has attracted considerable attention for its ability to reduce interfacial tension between immiscible fluids.^14–16^ Lower interfacial tension increases the capillary number, thereby enhancing the mobilization of residual oil trapped in pore spaces.^5,9,17^ This mechanism is essential for overcoming capillary forces that otherwise impede oil displacement, especially in water-wet formations.^18^

Surfactants reduce interfacial tension by virtue of their dual affinity for polar and nonpolar phases.^19^ In aqueous systems, they align at the oil–water interface, with hydrophilic heads in water and hydrophobic tails in oil.^20^ This arrangement weakens cohesive forces within each phase and relaxes interfacial stress, resulting in lower tension.^21^ Consequently, oil displacement is enhanced both at the pore scale and during larger-scale flow, improving overall sweep efficiency.^22,23^

Methyl ester sulfonate (MES) is an anionic biosurfactant derived from renewable resources like palm oil and is noted for its low manufacturing costs, high detergency with minimal dosage, biodegradability, and high tolerance to hard water.^24–26^ It also has various applications outside the EOR operation, such as in detergents and cleaning agents.^26^ MES is also known for its eco-friendly properties such as biodegradability and lower toxicity to the environment.^24,26,27^

This study aims to investigate the application of MES as a chemical EOR agent in spontaneous imbibition experiments. Our study aims to extend the application of MES for high salinity up to 700 mM NaCl or equivalent to 40 kppm and high-temperature (80 °C) conditions and for the crude oil from the Sumatran oil field in Indonesia. An earlier version of this work appeared in ref 28, where the coauthors had presented preliminary results (around 9% of the data shown in this research). This paper provides a significantly extended analysis and introduces new contents and findings: surface tension, modeling of surface and interfacial tensions, zeta potential, thermogravimetry, viscosity, contact angle, Winsor type, and normalized desaturation curve from an extensive database, and we also introduce a new definition of capillary number. This work also expands the preliminary limited cases into varied salinities (0, 500, 700 mM NaCl), surfactant concentrations (1 μM to 5 mM), and a comparison of two temperature settings (25 and 80 °C).

## 2 Methods

### 2.1 Materials

We use Berea sandstone as a representative sample of reservoir sandstone rock. The measurement by XRD (Empyrean diffractometer instrument at 40 mA and 45 kV) shows that more than 80% of the minerals comprise quartz. The rock samples are cut into substrates, and their surfaces are then smoothed by using sandpaper with a particle size of P500. The crude oil sample is obtained from one of the Sumatra oil fields in Indonesia. This oil sample is a light crude oil class with a value of 33.8 °API or equivalent to 0.856 g/mL at 25 °C. Oil viscosity is 62.8 cP at 25 °C and 40.3 cP at 80 °C. Detailed core and crude oil properties followed by each treatment are available in the Supporting Information.

Naturally, sodium (Na) acts as the counterion for MES and counters the negative ion on its oxygen atom, O (Figure 2). We use a 16-carbon MES, and thereby, we also write the MES surfactant as C_16_MES. The surfactant is prepared at a concentration of 5 mM and then diluted to lower concentrations, down to 1 μM. The synthetic saline water contains NaCl only with concentrations of 500 and 700 mM NaCl, or equivalent to 29 and 41 kppm total dissolved salt (TDS). Both saline solutions used as solvents for the C_16_MES surfactant, 500 and 700 mM NaCl, can be classified as highly saline and very-highly saline water, respectively, according to the salinity classification reported by refs 29 and 30 .

**Figure 2:**
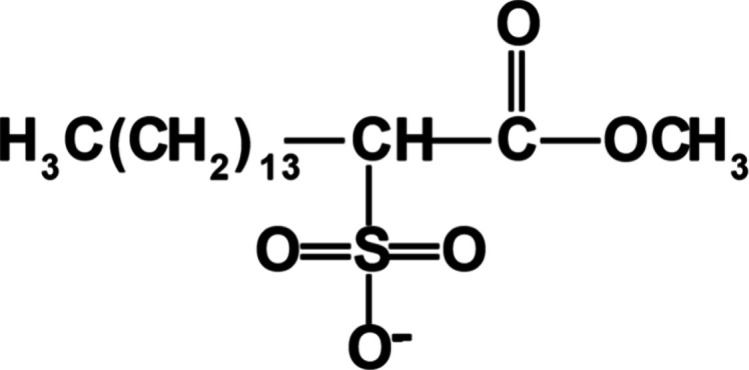
Methyl ester sulfonate (MES) chemical structure: C_16_ MES.

### 2.2 Characterizations

#### Surface and Interfacial Tensions

A surface tension (water–air interfacial tension) measurement with a Wilhelmy plate method (Kruss K100 tensiometer apparatus) is conducted at ambient pressure and a temperature of 25 °C (Figure 3), while the oil–water interfacial tension measurements use a spinning drop tensiometer (Kruss SDT) at both 25 and 80 °C (Figure 4). The trend of nonlinear decrease in surface and interfacial tension with increasing surfactant concentration is expressed by^31^

$$\sigma =\sigma_{\mathrm{CMC}}+(\sigma_{0}-\sigma_{\mathrm{CMC}})\mathrm{erfc}(\mathrm{KS})$$ 4

$$K=\frac{\sqrt{\pi }}{2S^{*}}\frac{\sigma_{0}}{\sigma_{0}-\sigma_{\mathrm{CMC}}}$$ 5

**Figure 3:**
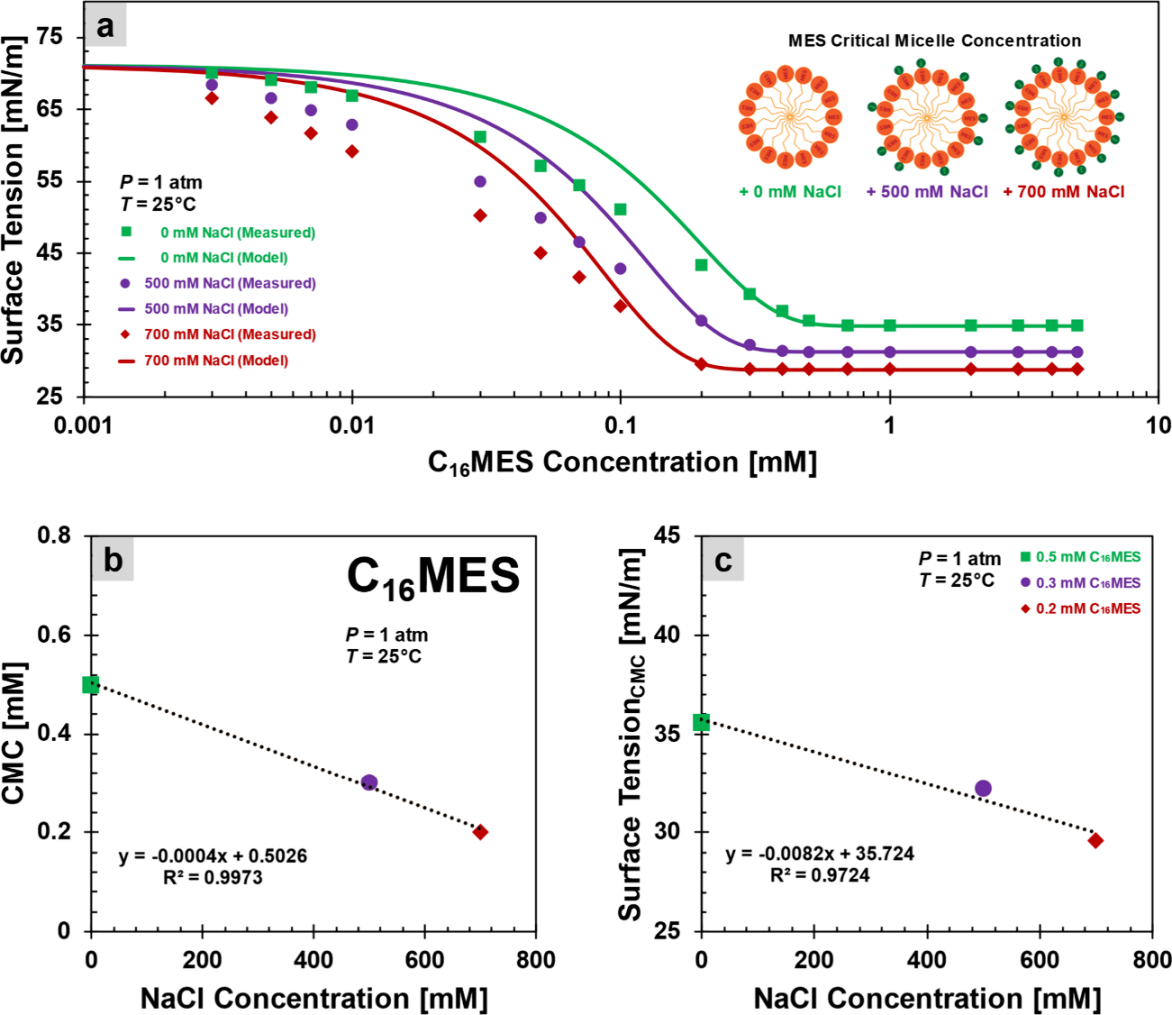
Surface tension. (a) Effect of increasing surfactant concentration on surface tension reduction. The lines are eq 4 with parameter σ_0_ as the initial surface tension and σ_CMC_ as the surface tension at critical micelle concentration; both are obtained from measurement. eq 4 is plotted with the green line (no salts): σ_0_ = 71 mN/m, σ_CMC_ = 35 mN/m, and *K* = 3.6; with the purple line (500 mM NaCl): σ_0_ = 71 mN/m, σ_CMC_ = 31 mN/m, and *K* = 5.7; and with the red line (700 mM NaCl): σ_0_ = 71 mN/m, σ_CMC_ = 28 mN/m, and *K* = 8.2. (b) Effect of increasing salt concentration on the critical micelle concentration (CMC) points: 0.2, 0.3, and 0.5 mM C_16_MES for 0, 500, and 700 mM NaCl, respectively. (c) Effect of increasing salt concentration on the surface tension at each critical micelle concentration point σ_CMC_.

**Figure 4:**
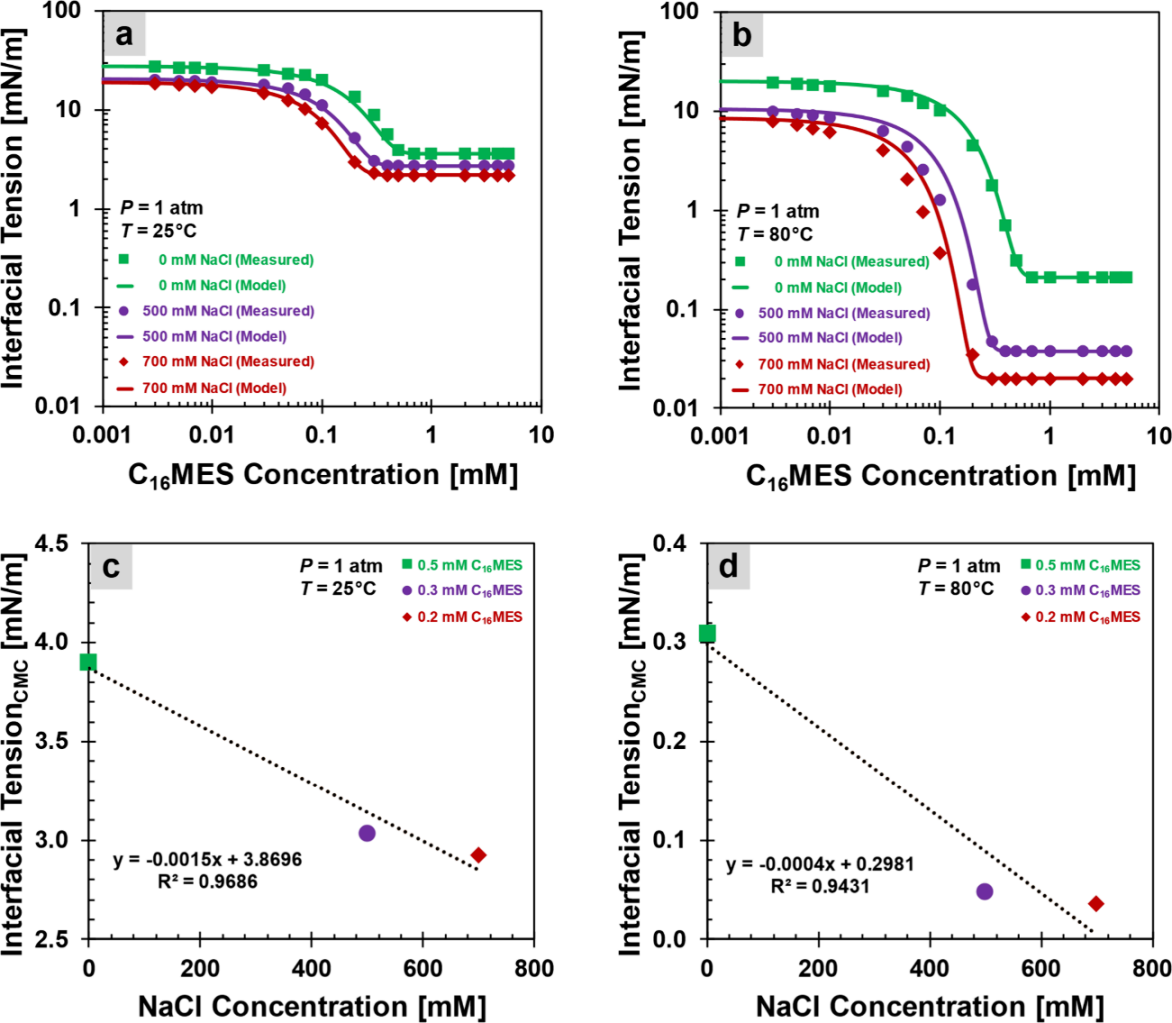
Interfacial tension, where the effects of temperature, surfactant, and salt concentrations on interfacial tension behavior are highlighted. (a) Effect of increasing surfactant concentration on interfacial tension at 25 °C. For the temperature of 25 °C, eq 4 is plotted with the green line (no salts): σ_0_ = 29 mN/m, σ_CMC_ = 3.6 mN/m, and *K* = 3.5; with the purple line (500 mM NaCl): σ_0_ = 21 mN/m, σ_CMC_ = 2.7 mN/m, and *K* = 5.5; and with the red line (700 mM NaCl): σ_0_ = 19 mN/m, σ_CMC_ = 2.2 mN/m, and *K* = 7. (b) Effect of increasing surfactant concentration on the interfacial tension at 80 °C. For the temperature of 80 °C, eq 4 is plotted with the green line (no salts): σ_0_ = 20 mN/m, σ_CMC_ = 0.2 mN/m, and *K* = 4; with the purple line (500 mM NaCl): σ_0_ = 11 mN/m, σ_CMC_ = 0.04 mN/m, and *K* = 8; and with the red line (700 mM NaCl): σ_0_ = 9 mN/m, σ_CMC_ = 0.02 mN/m, and *K* = 12. (c) Effect of increasing salt concentration on the interfacial tension at 25 °C. (d) Effect of increasing salt concentration on the interfacial tension at 80 °C.

In this model, σ_0_ and σ_CMC_ represent the interfacial tensions at zero and critical micelle concentration, respectively. The constant *K*, however, is treated as a fitting parameter and is not directly determined with eq 5. According to ref 31, eq 5 relates to an extrapolated concentration S*, which is obtained from the initial slope of the interfacial tension reduction curve. This adjustment allows the model to more accurately capture the experimental interfacial tension data, particularly in cases where early stage measurements make a reliable graphical estimation difficult.

#### Zeta Potential

We measure the zeta potential (ζ-potential) of aqueous surfactant-brine mixtures at varying surfactant concentrations and salinities using a dynamic light scattering-based zeta sizer (Figure 5). When mixed with oil, the surfactant-brine-oil system can form microemulsion structures, which indicates its potential for oil mobilization (Figure 6). The measurements are not intended to determine the zeta potential of colloidal solids or intact porous media, which are better characterized using a streaming potential setup (see refs 32,33 ). Instead, the zeta potential is measured on surfactant solutions without solid particles to evaluate the electrostatic behavior and colloidal stability of the system.

**Figure 5:**
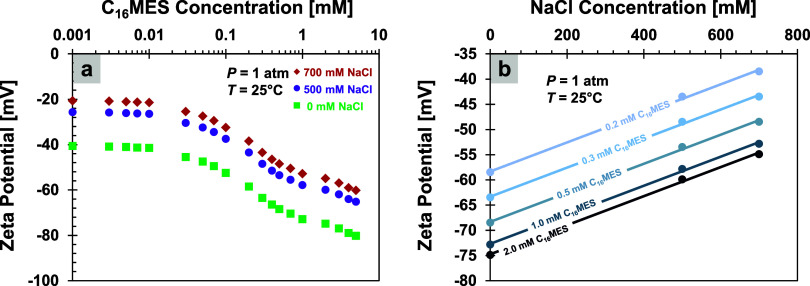
Zeta potential. (a) Effect of increasing surfactant concentration on the zeta potential. (b) Effect of increasing salt concentration on the zeta potential. Lines are only to guide the eyes, not based on an equation.

**Figure 6:**
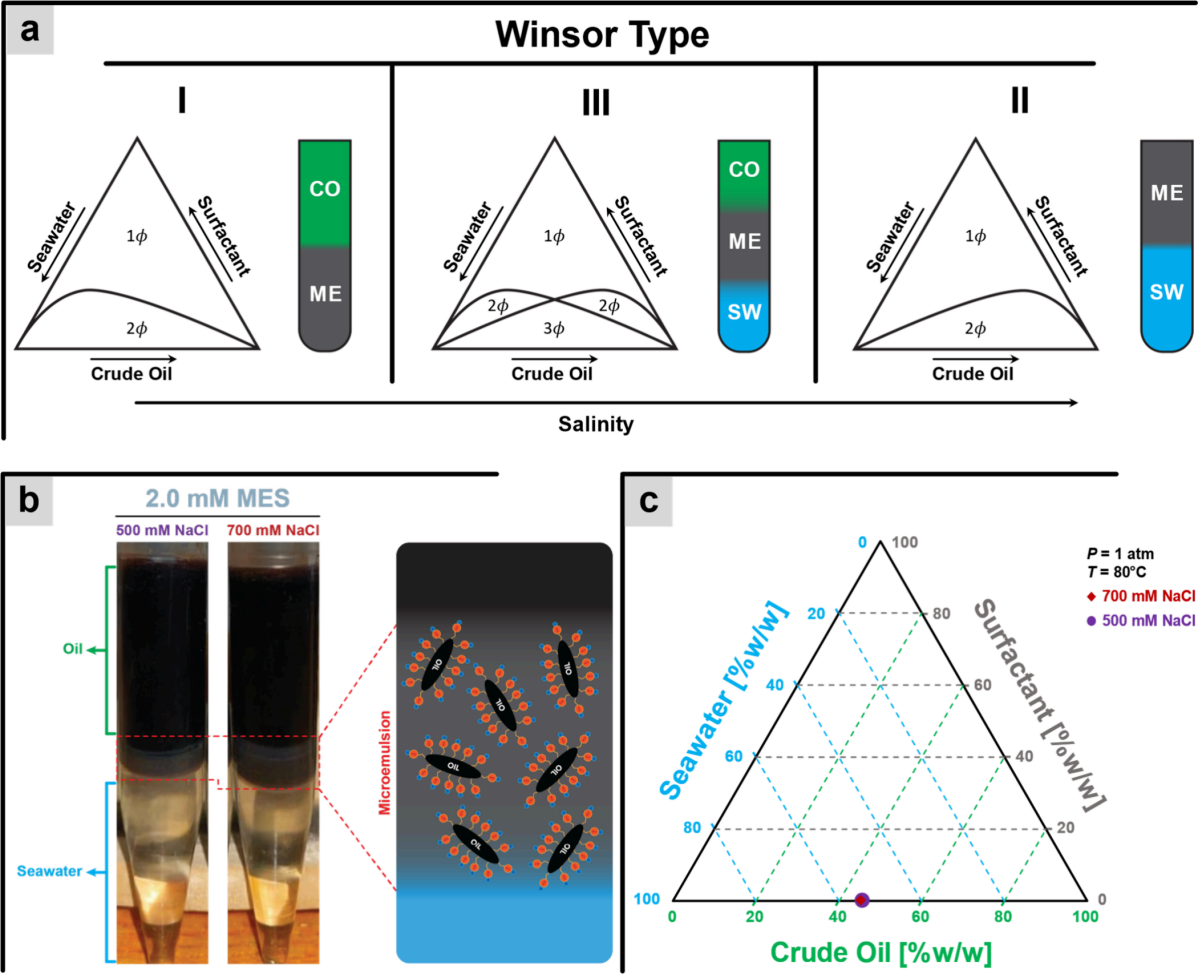
Winsor classification. (a) Effect of salinity on Winsor type. The symbol ϕ represents the number of phases. (b) MES microemulsion result. (c) Ternary diagram of Winsor-type MES. CO: Crude oil, SW: Seawater, ME: Microemulsion.

#### Thermal Analyses

Viscosity of samples is measured with an Ostwald viscosimeter whose instrument has a speed constant of 0.4994 St/s. We also measure the viscosity for each surfactant concentration at different salinities and temperatures (Figure 7a,b). We also perform an ambient pressure thermal analysis from 20 to 500 °C with a thermogravimetric analyzer (PerkinElmer—TGA 8000) to obtain the mass loss and stability (Figure 7c).

**Figure 7:**
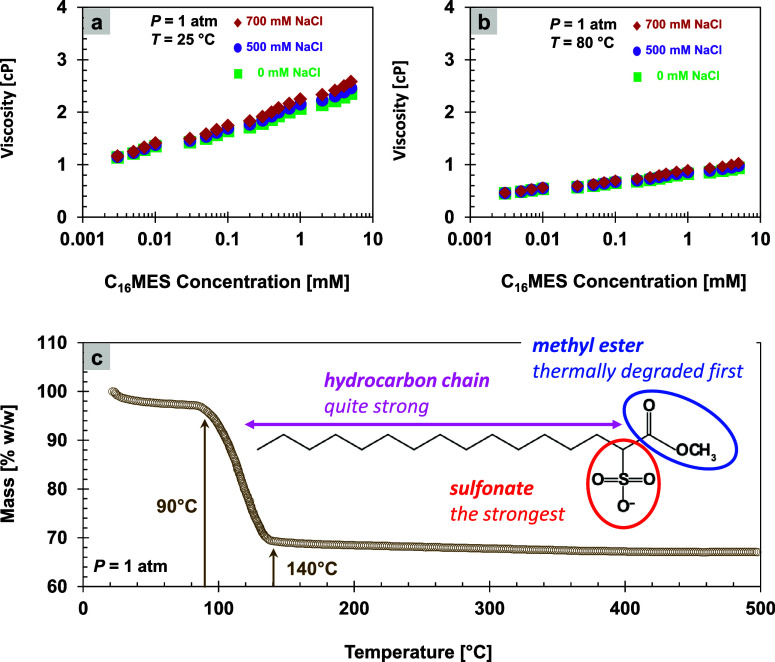
Thermal effects on viscosity and mass. (a) Effect of temperature on surfactant viscosity at 25 °C and (b) 80 °C. (c) Effect of increasing temperature on surfactant structure/mass.

#### Contact Angle

In the sessile drop method, a water droplet is placed on the rock surface, and the internal angle formed between the rock, water drop, and air is used to assess wettability.^34^ Contact angles are measured using a Ramé–Hart goniometer (sessile drop method) to monitor changes in rock wettability at initial (day 0), after 7, and 14 days. Sandstone samples are prepared by cutting the rock into several coin-shaped pieces, polishing the surfaces with fine sandpaper, and rinsing them with deionized water. The rock surfaces are manually polished using fine sandpaper to minimize irregularities and obtain a uniform texture before contact angle testing. This simple preparation method has been shown effective in reducing roughness-induced variations in wettability measurements.^35^ Consistent results are obtained from repeated measurements and confirmed that the surfaces are sufficiently smooth after polishing. The polished samples are then oven-dried for 7 days to remove residual moisture and then placed under vacuum for 1 day before saturation with crude oil for 2 weeks.

After the initial contact angle is confirmed with air as the surrounding phase, the oil-aged samples are immersed in the surfactant solution and aged in an oven at 80 °C. The second and third measurements are taken on the seventh and 14th days, respectively, also using air as the surrounding phase. All measurements are conducted at 1 atm and 25 °C after ensuring that the sample temperature has been equilibrated upon removal from the oven.

### 2.3 Spontaneous Imbibition

This study employs the Amott concept in a spontaneous imbibition experiment.^36^ All core samples undergo treatment simultaneously under identical temperature conditions at 80 °C. The experiment runs for 24 days, and each day, we record the volume of oil that rises to the top of each cell as recovered oil.

## 3 Results and Discussion

### 3.1 Salinity Effects

#### Salinity vs Interfacial Tension

Surface tension denotes the forces observed at the interface between air and water. Water prefers its own neighboring water molecules, and air prefers its air environment. The surfactant reduces surface tension because of its amphiphilic structure, i.e., it contains both a hydrophilic (water-attracting) head and a hydrophobic (water-repelling) tail. This unique structure allows it to accumulate at interfaces (such as the air–water interface) and disrupt the cohesive forces between water molecules.

Figure 3a demonstrates that an increase in surfactant concentration leads to a gradual decrease in surface tension σ, up to a threshold surface tension σ_CMC_ beyond which further addition of surfactant produces negligible surface tension changes. The minimum concentration at which this plateau surface tension σ_CMC_ occurs is known as the critical micelle concentration (CMC). In salt-free environments, strong electrostatic repulsion between surfactant molecules with uniformly charged hydrophilic heads inhibits micelle formation. Consequently, a no-salt case requires a higher surfactant concentration to reach the CMC.^37^

Adding salts such as NaCl introduces Na^+^ ions that interact with surfactant hydrophilic heads O^–^ and partially neutralize their charges through ion–ion or ion–dipole interactions.^38^ These interactions reduce intermolecular repulsion among surfactant molecules and promotes micelle formation at lower surfactant concentrations.^39^ Figure 3a,b displays a significant decrease in CMC and a more rapid drop in surface tension compared to those under salt-free conditions.

Salt ions not only screen (shield) electrostatic repulsion between charged surfactant head groups but also compress the electrical double layer. This promotes greater adsorption of surfactant molecules at the air–water interface, allowing them to pack more densely. As a result, higher salinity reduces the surface tension more effectively (Figure 3a) and also results in a lower surface tension at the critical micelle concentration (σ_CMC_), as shown in Figure 3c.

Similarly, the addition of a surfactant to an oil–water system causes the molecules to migrate to the interface, where they self-assemble to stabilize the boundary between the two immiscible phases. The hydrophobic tails orient toward the oil phase, while the negatively charged hydrophilic heads remain in the aqueous phase. This arrangement reduces the interfacial tension between the oil and water. Figure 4a,b illustrates the resulting decrease in oil–water interfacial tension, denoted by σ_ow_ at 25 and 80 °C. In some contexts, the symbol σ is used interchangeably to represent both surface and interfacial tensions, depending on the specific interface under discussion.

Salinity plays a role similar to that at this oil–water interface. Na^+^ ions present in seawater act as electrostatic shields by partially neutralizing the negative charges on the surfactant heads, thereby reducing intermolecular repulsion.^40^ This reduction allows surfactant molecules to pack more densely and adsorb at the oil–water interface, forming a more stable interfacial layer.^40^ Figure 4c,d depicts a more pronounced reduction in interfacial tension resulting from the denser surfactant packing.

Excessive salinity, particularly in the presence of divalent ions such as Ca^2^^+^ and Mg^2^^+^, can induce surfactant precipitation through the formation of insoluble salts.^41^ Consequently, surfactant molecules are removed from the bulk phase and become unavailable for adsorption at the oil–water interface. This absence markedly reduces their ability to lower interfacial tension and compromises the effectiveness of the surfactant system in enhancing interfacial stability.^42^

Although this study uses NaCl brine to establish a baseline under monovalent conditions, the effects of divalent ions such as Ca^2^^+^ and Mg^2^^+^ should be further examined. These ions are common in formation waters and may alter surfactant behavior through ion bridging, precipitation, or competitive adsorption. Future work should therefore assess the interfacial stability and efficiency of the C_16_MES surfactant in brines containing divalent cations to ensure its applicability under more realistic reservoir conditions.

#### Salinity vs Zeta Potential

Zeta potential ζ measurements show that the surfactant aqueous stability depends on both NaCl and surfactant concentrations. At concentrations near or above the CMC, surfactant molecules self-assemble into charged micellar aggregates that act as dispersed colloidal entities. Their electrophoretic mobility can therefore be measured as an apparent zeta potential under different salinity conditions. The measured ζ-potential represents the micellar surface potential, which reflects the distribution of charged head groups and counterions at the micelle–solution interface.

Figure 5a reveals that higher surfactant concentrations lower the zeta potential, indicating a stronger negative value because the surfactant is anionic with negative charge from oxygen in alkyl sulfonates—S­(O_2_)­O^–^ (see the chemical structure in Figure 2). This bigger magnitude zeta potential indicates a stronger repulsion force, and hence the stability is better.^43–45^

In contrast, increasing the salinity from 0 to 700 mM renders the zeta potential less negative in a linear relationship (Figure 5b), as Na^+^ ions bind to the surfactant’s hydrophilic head and reduce surface charges. Another possible mechanism as to why the zeta potential is less negative in more saline conditions is due to the compression of the electrical double layer (the Stern and diffuse double layers). Increasing the ionic strength *I* reduces the Debye length λ_D_ ( $\lambda_{D}\propto 1/\sqrt{I}$ in refs 32,36,46 ), thereby compressing the electrical double layer; the potential at the shear plane is therefore reduced (apparent zeta potential becomes less negative) even if the surface charge density is unchanged.^47^ The final mechanism involves salt-induced changes in solution pH and the associated shifts in acid–base surface equilibria.^48^ However, because the pH is not recorded during the measurements, we cannot determine whether the experiments are conducted under equilibrium conditions or subject to pH variations. Although the presence of salt reduces the electrostatic repulsion, a low concentration of surfactant even at 1 μM maintains the zeta potential within a stable range |ζ| > 20 mV. As a result, colloidal stability is preserved even under conditions of high salinity.

#### Salinity vs Emulsions

Salinity also affects the formation of a stable and efficient microemulsion.^49^ Microemulsions are formed from a mixture of oil, water, and surfactants that can be classified into three main Winsor types.^50^ Winsor I consists of crude oil and an oil-in-seawater microemulsion; Winsor II consists of seawater and a seawater-in-crude oil microemulsion; and Winsor III poses three phase substances consists of crude oil, seawater, and microemulsion as a separate middle phase between crude oil and seawater. Figure 6a details this classification.^51,52^

Optimum salinity conditions will form a Winsor type III microemulsion, which is considered the most ideal type for oil recovery operations due to its balanced phase behavior.^53^ Too low and/or too high salinity will lead to achieving Winsor type I and/or Winsor type II, which is not good for oil recovery.^54^ Achieving the optimum salinity ensures a balanced interaction between oil–water–surfactant, resulting in favorable phase distribution and maximum oil recovery efficiency.^55^

Figure 6b provides evidence that MES can interact with oil and seawater to form microemulsions in both salinity conditions, 500 and 700 mM NaCl. The difference in microemulsion volume is indicated by distinct thicknesses of the middle phase. The thicker middle phase in the system with 700 mM salinity compared to 500 mM suggests that this may be the optimum salinity for the surfactant. However, higher salinity may shift the system to Winsor type II, which is unfavorable for oil recovery.

The Winsor type of the MES–oil–seawater system is depicted in Figure 6c. It is evident that the microemulsion formed corresponds to Winsor type III, as the experimental data falls within the three-phase region, consistent with the literature.^53^ This result clearly indicates that MES is capable of forming a microemulsion and maintaining its stability, even when exposed to high-salinity conditions.

### 3.2 Temperature Effects

#### Molecular Kinetics

The rising system temperature increases the kinetic energy of molecules in the solution, including surfactant molecules.^56,57^ This enhanced Brownian motion accelerates the mobility and diffusion of the surfactant to the oil–water interface, allowing the molecules to assemble more rapidly at the interface.^58–60^ The trends observed in Figure 4a,b indicate that an increasing temperature enhances the efficiency of surfactant adsorption at the oil–water interface. This improved adsorption more effectively disrupts cohesive interactions within both the oil and water phases, thereby leading to a further reduction in interfacial tension.

The CMC is relatively less sensitive to temperature changes depending on the surfactant type (Figure 4a vs Figure 4b). The lowest interfacial tension σ_CMC_, typically observed at CMC, is 1 order of magnitude lower at three times higher temperatures (Figure 4c vs Figure 4d). However, salinity plays a more prominent role in shifting the CMC, particularly for ionic surfactants, due to its effect on electrostatic interactions.

Figure 7a,b unveils that the temperature rise also lowers the viscosity of the surfactant solution. Increased molecular kinetic energy weakens intermolecular interactions such as van der Waals forces between hydrocarbon chains. Consequently, surfactant molecules move more freely. In addition, higher temperature accelerates molecular diffusion and reduces cohesion in the solution, which collectively decreases flow resistance.

#### Thermal Stability

High temperatures also pose risks. Many surfactants have solubility limits that temperature sensitively affects.^61^ At a certain point, decreasing surfactant solubility and compatibility in water causes some surfactants to become inactive at the oil–water interface.^61^ Furthermore, elevated temperatures disrupt micelle stability, increase the CMC, and can even lead to chemical degradation of the surfactant structure.^62–65^ High temperatures diminish the effectiveness of interfacial tension reduction, compromising the primary objective of using surfactants in EOR operations.^53^ Therefore, engineers must carefully evaluate both the high-temperature stability and long-term exposure durability.

Figure 7c describes the surfactant thermal stability up to 90 °C, making it well-suited for typical reservoir temperature conditions. However, when the temperature exceeds this threshold, degradation or volatilization may begin to occur. Therefore, extra caution is required when operating above 90 °C, and particularly beyond 140 °C, to ensure that performance is maintained and the material’s integrity is preserved.

The thermal stability of the surfactant is limited by the vulnerability of its ester group (−COO^–^) to heat,^66^ which causes partial decomposition at temperatures between 90 and 140 °C. Once these weaker components degrade, the molecule becomes thermally stable due to the presence of stronger groups such as sulfonates and long hydrocarbon chains.^67^ Typical degradation onsets occur for the ester group at approximately 150–250 °C, the hydrocarbon chain at 250–350 °C, and the sulfonate group at >350–450 °C. This signifies the initial mass loss, followed by stability at higher temperatures in the thermogravimetric analysis curve.

### 3.3 Wettability Alterations

#### Contact Angle Correlations

Contact angle θ is the main parameter to measure the wettability of a surface.^68–70^ A decrease in cos θ_ow_ corresponds to an increase in oil–water contact angles (Δθ_ow_ > 0) or a decrease in water–air contact angles (Δθ_wa_ < 0; see Figure 8). These changes reflect a transition toward a more favorable wettability condition for EOR, characterized by reduced oil-wetness or increased water-wetness of the surface. Although contact angles measured in water–air (wa) systems are less representative than those obtained from direct oil–water (ow) experiments, previous research has demonstrated a strong quantitative correlation between the two (Figure 8a,b):^71^

$$\cos(\theta_{\mathrm{wa},\mathrm{int}})=\left[\frac{1-\left(\frac{\sigma_{\mathrm{oa}}-\sigma_{\mathrm{ow}}}{\sigma_{\mathrm{wa}}}\right)}{2}\right]\cos(\theta_{\mathrm{ow},\mathrm{ext}})+\left[\frac{1+\left(\frac{\sigma_{\mathrm{oa}}-\sigma_{\mathrm{ow}}}{\sigma_{\mathrm{wa}}}\right)}{2}\right]$$ 6

where subindices “int” and “ext” denote internal and external angles, respectively, and subindices “oa,” “ow,” and “wa” designate oil–air, oil–water, and water–air interfacial tensions σ measured on a surface. Given θ_ow, int_ = 180° – θ_ow, ext_ and cos­(θ_ow, ext_) = – cos (θ_ow, int_), we can transform eq 6 to be

$$\cos(\theta_{\mathrm{ow},\mathrm{int}})=\left[\frac{-2}{1-\left(\frac{\sigma_{\mathrm{oa}}-\sigma_{\mathrm{ow}}}{\sigma_{\mathrm{wa}}}\right)}\right]\cos(\theta_{\mathrm{wa},\mathrm{int}})+\left[\frac{1+\left(\frac{\sigma_{\mathrm{oa}}-\sigma_{\mathrm{ow}}}{\sigma_{\mathrm{wa}}}\right)}{1-\left(\frac{\sigma_{\mathrm{oa}}-\sigma_{\mathrm{ow}}}{\sigma_{\mathrm{wa}}}\right)}\right]$$ 7

**Figure 8:**
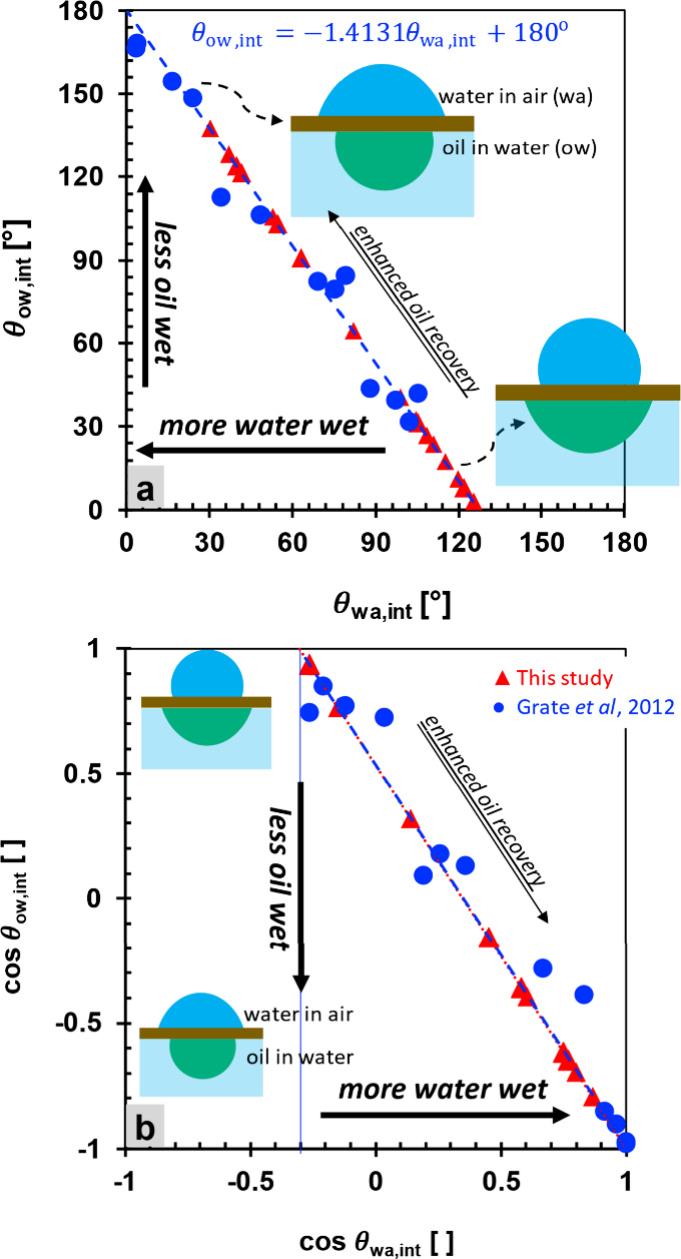
Correlation between water–air and oil–water contact angles. (a) Correlation of the internal water-in-air θ_wa,int_ and internal oil-in-water θ_ow_ contact angles on silanized silica and sandstone surfaces. (b) Cosine of the corresponding angles. Our study only measures water–air contact angles θ_wa, int_. Conversions into equivalent oil–water contact angles θ_ow, int_ use eq 7. The original data (Grate et al., 2012, in ref 71) presents the external oil–water angle θ_ow, ext_ being converted to internal angles: θ_ow, int_ = 180° – θ_ow, ext_.

The correlation line passes through the coordinate (cos θ_wa, int_, cos θ_ow, int_) = (1,–1), which represents a fully water-wet condition (Figure 8b). Under strongly oil-wet conditions (cos θ_ow, int_ = 1), the corresponding $\cos\;\theta_{\mathrm{wa},\mathrm{int}}=\frac{\sigma_{\mathrm{oa}}-\sigma_{\mathrm{ow}}}{\sigma_{\mathrm{wa}}}$ . Accordingly, hexadecanes on silanized silica surfaces exhibit cos θ_wa, int_ ≈ −0.3, consistent with the theoretical $\cos\;\theta_{\mathrm{wa},\mathrm{int}}=\frac{\left(\sigma_{\mathrm{oa}}-\sigma_{\mathrm{ow}}\right)}{\sigma_{\mathrm{wa}}}=-0.337$ , given IFTs:^71^ σ_oa_ = 27.5 mN/m, σ_ow_= 52.0 mN/m, and σ_wa_= 72.8 mN/m. Meanwhile, our crude oil and Berea sandstones exhibit $\cos\;\theta_{\mathrm{wa},\mathrm{int}}=\frac{\left(\sigma_{\mathrm{oa}}-\sigma_{\mathrm{ow}}\right)}{\sigma_{\mathrm{wa}}}=-0.303$ and θ_wa, int_ = 107.6°, resulting from given IFTs: σ_oa_ = 30.5 mN/m (calculated in the Supporting Information), σ_ow_ = 52 mN/m,^71^ and σ_wa_ = 71 mN/m (σ_0_ at 25 °C in Figure 3a). We cannot use σ_ow_ from σ_0_ in Figure 4a because the σ_ow_ in eq 7 refers to a reverse oil droplet on a solid surface within a water environment (cf. ref 13), whereas the data in Figure 4a correspond to an oil droplet floating in water without contact with a solid surface (spinning drop method).

#### Aging and Roughness

The aging process primarily alters the surface chemistry and wettability of rocks,^72–74^ although in some cases, it can also induce minor morphological changes due to mineral dissolution or precipitation, depending on the composition of the aging fluid and the mineralogy of the rock.^75^ An earlier study shows that surfaces with roughness from 17 to 943 nm exhibit 53° to 33° on contact angle variations.^35^ Another study reveals that roughness-induced wettability alteration is mainly linked to clay swelling, with greater contact angle shifts in clay-rich sandstones.^13^ In this study, the Berea sandstone contains only minor clay minerals: 3.2% muscovite, 4.2% kaolinite, and 0.3% illite. Both muscovite and kaolinite are nonswelling minerals with low specific surface areas, while the illite fraction is negligible. Rocks with specific surface area *S*_*S*_ < 3 m^2^/g are silty to nonclayey and exhibit low surface reactivity.^32,76^ Therefore, the influence of roughness evolution during aging is expected to be minimal, unlikely to alter the wettability, and the initial wettability originates from oil adsorption rather than morphological changes.

Nevertheless, the influence of surface roughness and aging time on wettability, although well recognized, remains not yet fully understood.^77^ Therefore, this work acknowledges the absence of quantitative surface roughness characterization, such as using atomic force microscopy or scanning electron microscopy. Future studies are recommended to employ these techniques to better quantify surface morphology evolution and its potential contribution to wettability alterations.

#### Surfactant Effects

Surfactants significantly alter sandstone wettability in saline environments by changing rock–fluid interactions.^78^ Surfactant treatment transforms initially oil-wet sandstone into water-wet sandstone.^79^ Figure 9 expresses that higher surfactant concentrations improve wettability alteration, as shown by reduced inner contact angles. Additionally, increasing NaCl salinity slightly decreases the efficiency of wettability alteration.^80,81^

**Figure 9:**
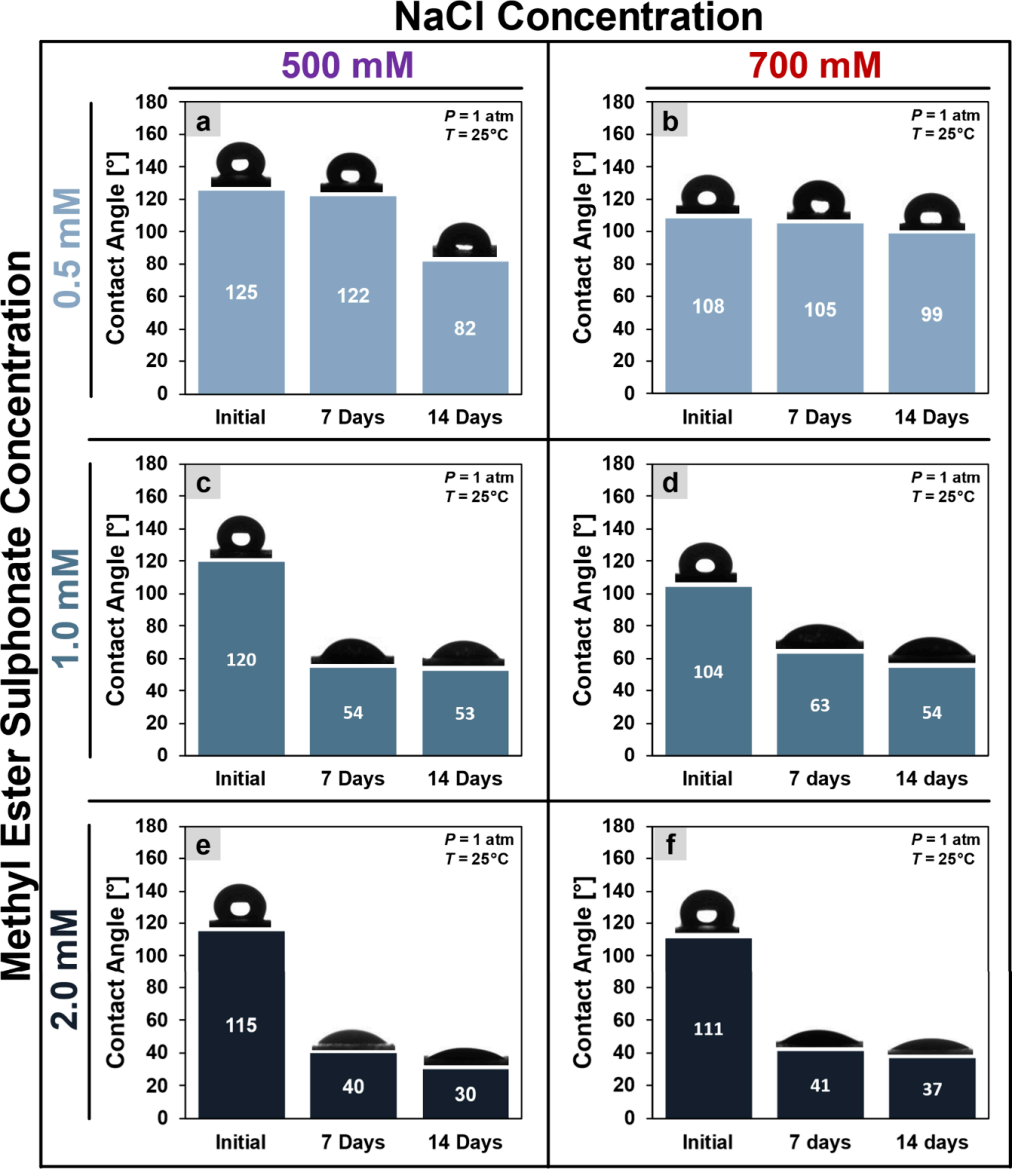
Contact angle. Inner contact angle of a water droplet on sandstone rocks treated by MES. Columns indicate 500 mM salinity (a,c,e) and 700 mM (d,e,f). Rows indicate surfactant concentrations in mM: 0.5 (a,b), 1 (c,d), and 2 (e,f).

#### Oil Desorption Mechanisms

The change in wettability occurs primarily through the desorption of the oil layer from the sandstone surface. At the same time, the position vacated by the desorbed oil on the rock surface is gradually replaced by some Na^+^ ions present in seawater. Additionally, other Na^+^ ions interact and bind with the hydrophilic head groups of the surfactants, further influencing the wettability alteration process.

The basic and acidic groups in the oil attach to the sandstone surface through a combination between van der Waals and Coulombic forces,^82^ driven by differences in electrical charge, as Figure 10a,d portrays. At pH ≥ 7, the oil interface would mainly be [>R−COO^–^] instead of [>R−NH^+^].^36,83^ When the surfactant is injected, it disrupts the forces between the sandstone surface and the oil layer.

**Figure 10:**
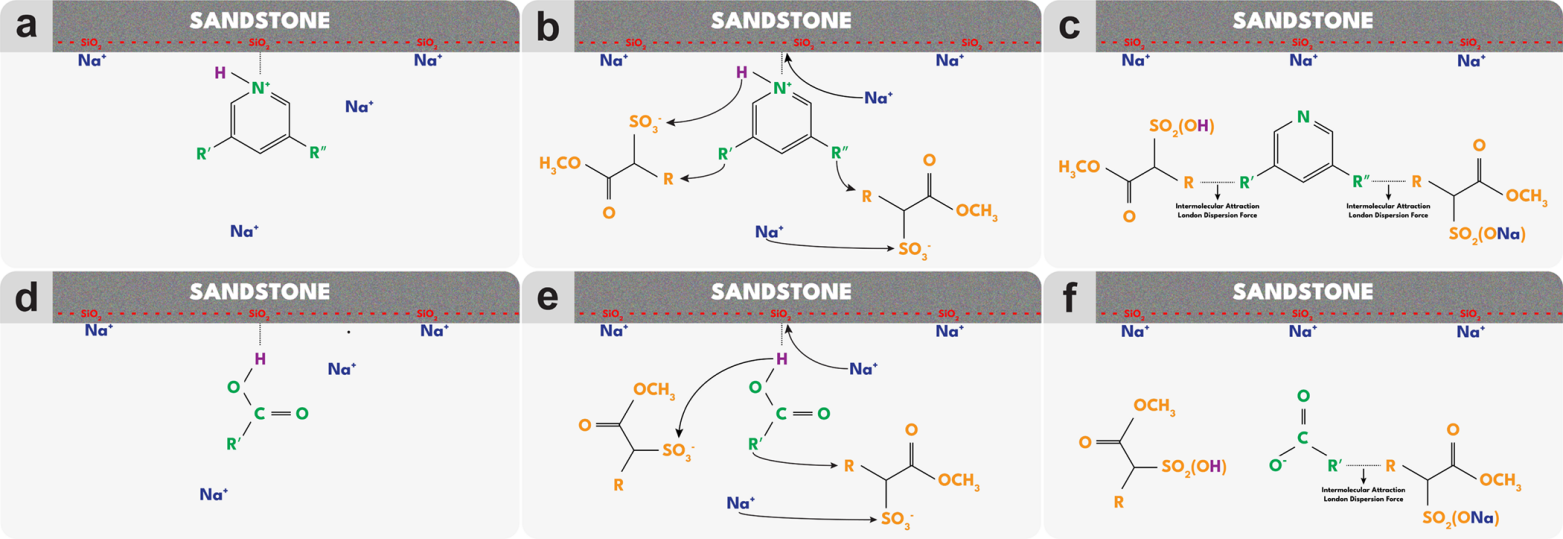
Mechanisms of surfactant to enhance oil desorption from sandstone. Crude oil exhibits polar functionality: ≡NH^+^ for (a–c); and – COO^—^ for (d–f).

In acidic environments, the alkyl sulfonate attracts crude oil’s ≡NH^+^ so that the proton (H) is released from the oil (Figure 10b). The uncharged oil molecules interact with the hydrophobic tails of the surfactant through London dispersion forces (a type of van der Waals force), facilitating the incorporation of the oil phase into micelles or emulsions through solubilization (Figure 10c). While London dispersion forces are individually weak, their collective effect in micelles can be significant, particularly for trapping and mobilizing oil in EOR.

At neutral or alkaline pH, acidic components in crude oil (such as −COOH) can deprotonate to form negatively charged carboxylates (−COO^–^; see Figure 10e). These negatively charged oil components can then be rejected by negatively charged rock surfaces (Figure 10f). This repulsion increases the electrostatic force at the rock–oil interface and thickens the electric double layer. Figure 10c,f elucidates the desorption of the oil layer from the sandstone surface. As a result, the sandstone repels the oil and becomes more water-wet.

### 3.4 Oil Recovery vs Capillary Number

The recovery factor gradually approaches a plateau after around 14 days, indicating that the imbibition process has nearly reached equilibrium, and no further oil could be spontaneously displaced from the core by the surfactant (compare Figures 11 and S1 in the Supporting Information). This also suggests that the surfactant retains its interfacial activity under high-temperature and high-salinity conditions over an extended period (2 weeks).

**Figure 11:**
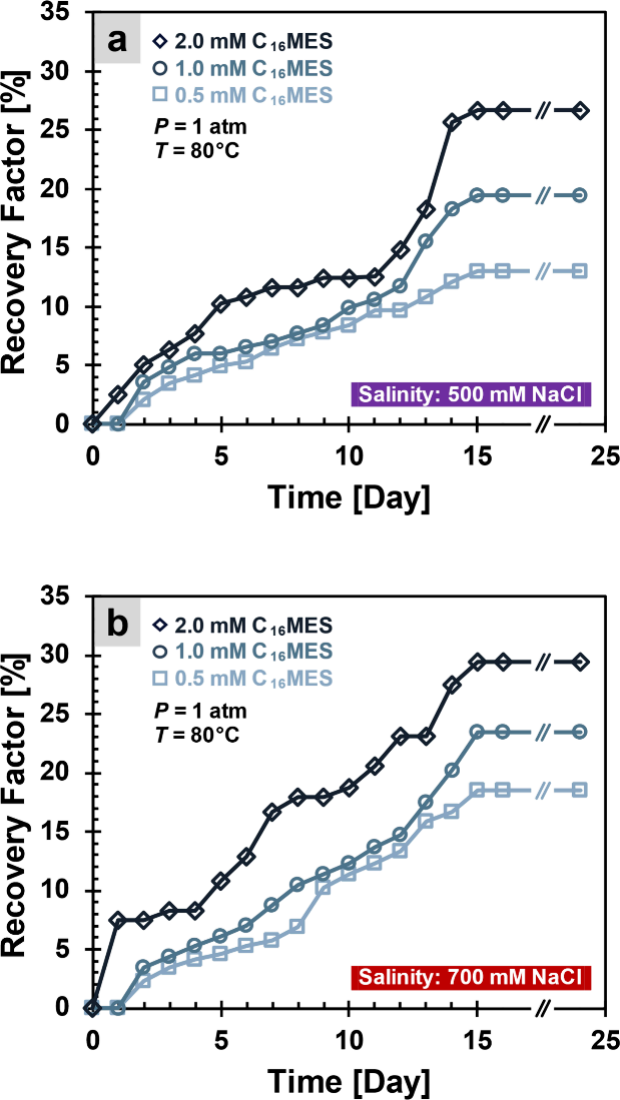
Oil recovery factor. Oil recovery through a spontaneous imbibition experiment with the surfactant. (a) Salinity conditions of 500 mM NaCl. (b) Salinity conditions of 700 mM NaCl. For extended data for days 15–25, please refer to Supporting Information: Figure S1.

Figure 11 clearly shows that increasing the surfactant concentration consistently improves the recovery factor under both salinity conditions. At 500 mM salinity, the 2.0 mM surfactant concentration achieves the highest recovery, about 27% after 14 days, while 1.0 and 0.5 mM concentrations resulted in progressively lower recoveries (Figure 11a). Similar trends are observed at 700 mM salinity, where 2.0 mM again produces the highest recovery, close to 28% (Figure 11b).

Thus, increasing the surfactant concentrations induces more oil recovery due to the decreased oil–water interfacial tension (Figure 4) and altered wettability toward a more water-wet condition (Figure 9). A higher surfactant concentration also increases the viscosity of the aqueous system, thereby potentially enhancing the sweep efficiency if applied in a flooding system rather than in a spontaneous imbibition setting.

Interestingly, when compared between the two salinities, the recovery factors are generally greater at higher salinities for each of the same surfactant concentrations (Figure 11). This observation suggests that increasing salinity may strengthen the spontaneous imbibition mechanism, possibly through more reductions on interfacial tension (Figure 4), and consequently the capillary forces that govern fluid displacement within porous media.

To elucidate the reason for the higher oil recovery observed in the 700 mM NaCl case (Figure 11), the capillary number *N*_Ca_ is compared between the 500 and 700 mM NaCl systems. The typical invading fluid velocity *v*_inv_ of water–surfactant flooding fronts in oil reservoirs, such as during secondary recovery, is relatively low, ranging from 10^–7^ to 10^–4^ m/s (equivalent to 0.01–10 m/day).^5,84,85^ The value of *v*_inv_ derived from imbibition velocity *v*_imb_ at 10^–9^ m/s is considered in the calculation of *N*_Ca_ (eq 2). As expected, imbibition occurs much more slowly than flooding (the calculation of *v*_imb_ is provided in the Supporting Information). Figure 8b shows that cos­(θ_ow, int_) spans from −1 to 1; therefore, we define a new expression for *N*_Ca_ with the term (1 + cos θ_ow_) in the denominator. Adding 1 shifts the curve in Figure 8b vertically, resulting in strictly positive values. This form is suitable for representing the data on a logarithmic scale in the capillary desaturation curve (Figure 1). In the case of the 2 mM surfactant, the capillary number ratio $r_{\mathrm{Ca}}=\frac{N_{\mathrm{Ca}|\mathrm{final}}}{N_{\mathrm{Ca}|\mathrm{initial}}}$ is 4862 for 500 mM NaCl and 5230 for 700 mM NaCl. Larger values of the capillary number ratio *r*_Ca_ correspond to greater oil recovery.

Each rock sample has distinct irreducible water *S*_wi_ or residual oil saturation *S*_or_ as their minimum saturation, i.e., *S*_wi, min_ or *S*_or, min_. Therefore, we want to generalize them into normalized residual saturation *S*_*n*_ which we compute with the following formulation:

$$S_{n}=\frac{S_{\mathrm{or}}-S_{\mathrm{or},\min}}{S_{\mathrm{or},\max}-S_{\mathrm{or},\min}}$$ 8

for a given residual oil saturation *S*_or_ and its known minimum *S*_or, min_ and maximum *S*_or, max_ values. Remarkably, the capillary desaturation curves reveal that normalized residual fluid saturation *S*_*n*_ ranging from 1 to 0 is achievable by increasing the capillary number *N*_Ca_ by 2-to-4 orders of magnitude or equivalently by raising the capillary number ratio *r*_Ca_ by a factor of 10^2^-to-10^4^. We mention “fluid” in normalized residual fluid saturation *S*_*n*_ because the flooding experiment data are not only from the oil displacements but also from brine and microemulsions. The previously compiled data in Figure 1 also support the findings in our experimental data.

Overall, the experimental results demonstrate that increasing both the surfactant concentration and the solution salinity significantly enhances oil recovery efficiency during spontaneous imbibition when using the MES surfactant. Extending oil recovery experiments from static spontaneous imbibition to dynamic displacement methods (such as core flooding and milli- or microfluidic models) would enable a more comprehensive evaluation of the surfactant’s performance under the combined influence of capillary *F*_c_ and viscous *F*_v_ forces. Such investigations would bridge the gap between laboratory-scale observations and field-scale applications, thereby providing essential data for simulation and scale-up.

## 4 Conclusions

The potential of methyl ester sulfonate (MES) for surfactant-based oil recovery has been evaluated through various tests under high salinity (up to 700 mM NaCl) and high temperature (80 °C). Key findings from surface and interfacial tensions, zeta potential, thermal stability, viscosity, wettability, and spontaneous imbibition tests are as follows.

- MES surfactants reduce interfacial tensions down to 0.02 mN/m (at 80 °C). Salinity (up to 700 mM NaCl) shifts the critical micellar concentration lower to 0.2 mM through the charge-screening effect of Na^+^ ions, promoting surfactant packing and adsorption. Although higher salinity makes the zeta potential less negative, all studied ranges of surfactant concentrations (1 μM–5 mM) maintain colloidal stability. The aqueous salinity also promotes Winsor type III microemulsion formation and is effective for oil mobilization.
- At a high temperature (80 °C), the surfactant exhibits improved performance due to faster diffusion and adsorption at the oil–water interface, leading to quicker interfacial tension reduction. Higher temperatures also decrease oil viscosity, enhancing fluid mobility. Thermogravimetric analysis shows the surfactant is stable up to 90 °C, with partial decomposition between 90 and 140 °C due to ester group breakdown. Beyond this, sulfonate and hydrocarbon chains provide stability. Thus, the surfactant performs well under typical reservoir temperatures but requires caution above 140 °C.
- Contact-angle analyses confirm that the surfactant induces a transition from preferentially oil-wet to preferentially water-wet conditions via combined electrostatic repulsion and solubilization of surface-bound oil. The observed behavior aligns with desorption mechanisms involving Coulombic and London dispersion interactions at the rock–fluid, oil–brine, and oil–surfactant interfaces.
- We propose a new definition of capillary number $N_{\mathrm{Ca}}=\frac{\mu_{\mathrm{inv}}v_{\mathrm{inv}}}{\sigma_{\mathrm{ow}}(1+\cos\;\theta_{\mathrm{ow}})}$ where the value is strictly positive due to the inclusion of the shifting term (1 + cos θ_ow_). The viscosity μ_inv_ and velocity v_inv_ of the invading fluid are parameters of the surfactant-brine system, whereas the oil–water interfacial tension σ_ow_ is more accurately determined from measurements on a solid surface, where three-phase interfacial forces are present.
- An increase in the capillary number by 2-to-4 orders of magnitude results in greater oil recovery, reaching the residual oil saturation. Increased capillary numbers are due to corroborating factors from higher surfactant concentrations and salinities that yield lower oil–water interfacial tensions and alter the sandstone wettability to be preferentially water-wet. In the end, MES demonstrates suitability for field implementation in oil reservoirs with similar characteristics.

## Supplementary Material



## Data Availability

Data for the main figures (in .xlsx format) presented in the manuscript are also available in the NYCU repository: 10.57770/EVYSSU.

## Notes

Detailed mineral compositions, crude oil, surfactant mixtures, core sample properties, and imbibition profiles (recovery factor, flow rate, and imbibition velocity) (PDF)
