# Supplementary material for: Chemical EOR with Methyl Ester Sulfonate: Achieving Residual Oil Saturation via 2–4-Order Capillary Number Increase
Source: ACS Phys Chem Au. 2025 Dec 17;6(2):272–85. doi: 10.1021/acsphyschemau.5c00087 (PMC13022728; doi:10.1021/acsphyschemau.5c00087)
Supplement: Supplementary file 1 [file pg5c00087_si_001.pdf]

SUPPORTING INFORMATION

Chemical EOR with Methyl Ester Sulfonate: Achieving Residual Oil Saturation via 2−4-Order Capillary Number Increase

Authors:

Farizal Hakiki [1,a,b,#,*], Muhamad Raihan Al Fikri [c,#], Veni Dwi Amelia Putri [c], Indra Gunawan [c], Witta Kartika Restu [d], Muslim Abdurrahman [c]

Notes:

[1] legal name: Farizal Hakiki Soemarsono

[#] These authors contributed equally: FH & MRAF

Affiliations:

[a] National Yang Ming Chiao Tung University (NYCU), Disaster Prevention & Water Environment Research Center (DPWE), Hsinchu 300, Taiwan

[b] National Yang Ming Chiao Tung University (NYCU), Civil Engineering Department, Hsinchu 300, Taiwan

[c] Universitas Islam Riau, Engineering Faculty, Department of Petroleum Engineering, Pekanbaru, Riau 28284, Indonesia

[d] National Research and Innovation Agency (BRIN), Research Center for Chemistry, Serpong, South Tangerang 15314, Indonesia

Corresponding author:

* F. Hakiki Soemarsono (email: hakiki@nycu.edu.tw; alhakiki@live.co.uk)

Data for the main figures (in .xlsx format) presented in the manuscript are also available in the NYCU repository: https://doi.org/10.57770/EVYSSU

Table S 1. Mineral compositions of Berea sandstone

Minerals	Composition [%]
Quartz	80.2
Microline	7.4
Albite	4.6
Kaolinite	4.2
Muscovite	3.2
Ankerite	0.4

Table S 2. Crude oil properties

Properties	Magnitude
Viscosity μ (at 25 °C)	62.8 cP
Viscosity μ (at 80 °C)	40.3 cP
Density ρ (at 25 °C)	0.856 g/mL
Gravity γ^o (at 25 °C)	33.8 °API
Class	Light Crude Oil
Oil-Air Interfacial Tension* σ_{oa}	30.5 mN/m

*Calculation method (at 25 °C): $\sigma_{\mathrm{oa}} = 39.22 - 0.258\gamma^o$, fitting based on database in ref [1]

Table S 3. Detailed methyl ester sulfonate and salinity mixtures

Surfactant Formulations	MES Concentration	NaCl Salinity
Formulation #1	0.5 mM	500 mM
Formulation #2	2.0 mM	500 mM
Formulation #3	3.0 mM	500 mM
Formulation #4	0.5 mM	700 mM
Formulation #5	2.0 mM	700 mM
Formulation #6	3.0 mM	700 mM

Table S 4. Core sample properties

Properties	Core					
	#1	#2	#3	#4	#5	#6
Diameter d [cm]	3.2	3.2	3.2	3.2	3.2	3.2
Length l [cm]	4.5	4.4	4.6	4.6	4.4	4.4
Bulk volume V_b [mL]	36.19	35.39	36.19	37.00	36.19	35.39
Pore volume V_p [mL]	6.91	6.65	6.84	6.88	6.73	6.65
Oil volume at initial V_{oi} [mL]	6.91	6.65	6.84	6.88	6.73	6.65
Porosity ϕ [%]	19.1	18.8	19.0	18.6	18.2	18.8
Initial oil saturation S_{oi} [%]	99.99	99.99	99.99	99.98	99.99	99.99

Table S 5. Spontaneous imbibition experiment results

Samples		V_{oi}	V_r	RF
Rocks	Surfactant Formulations	[mL]	[mL]	[%]
Core #1	Formulation #1	6.91	0.90	13
Core #2	Formulation #2	6.65	1.30	19
Core #3	Formulation #3	6.84	1.83	27
Core #4	Formulation #4	6.88	1.27	18
Core #5	Formulation #5	6.73	1.58	23
Core #6	Formulation #6	6.65	1.96	29

Note: V_{oi} is initial oil volume, V_r is recovered oil volume, and RF stands for the recovery factor.

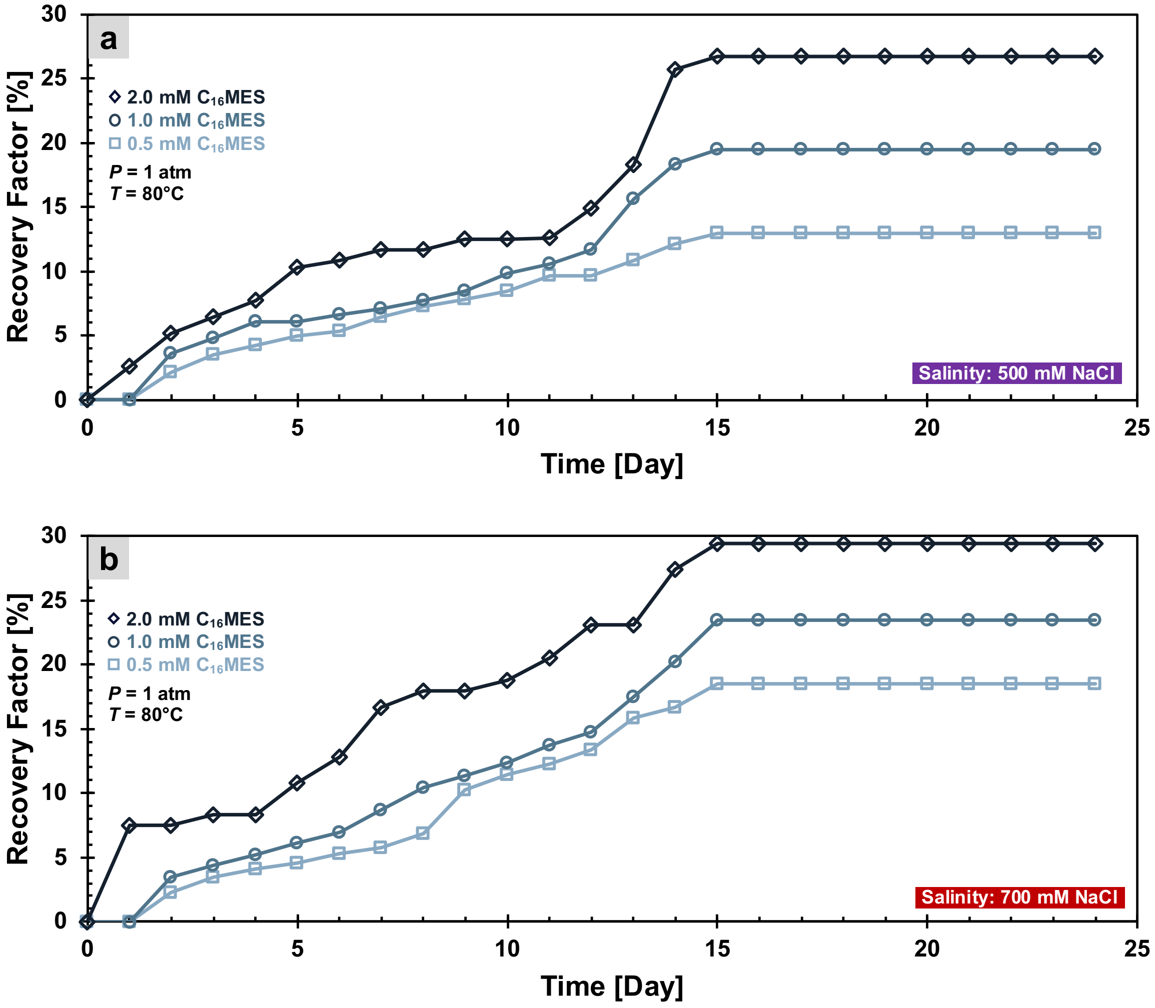

Figure S 1. Recovery factor profile from day 0 to 24. (a) Salinity at 500 mM NaCl and (b) 700 mM NaCl.

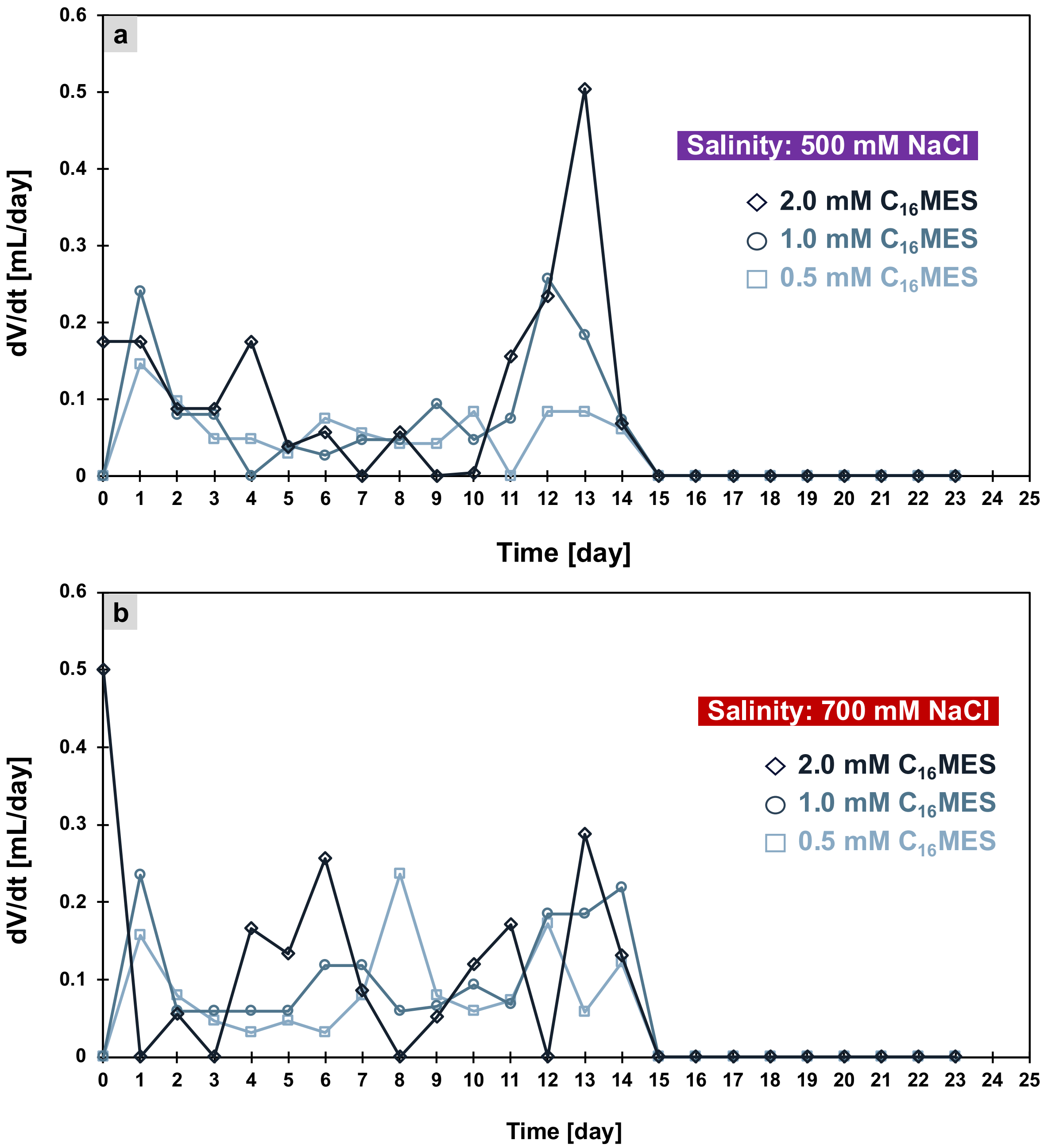

Figure S 2. Rate dV/dt profile from day 0 to 24. (a) Salinity at 500 mM NaCl and (b) 700 mM NaCl.

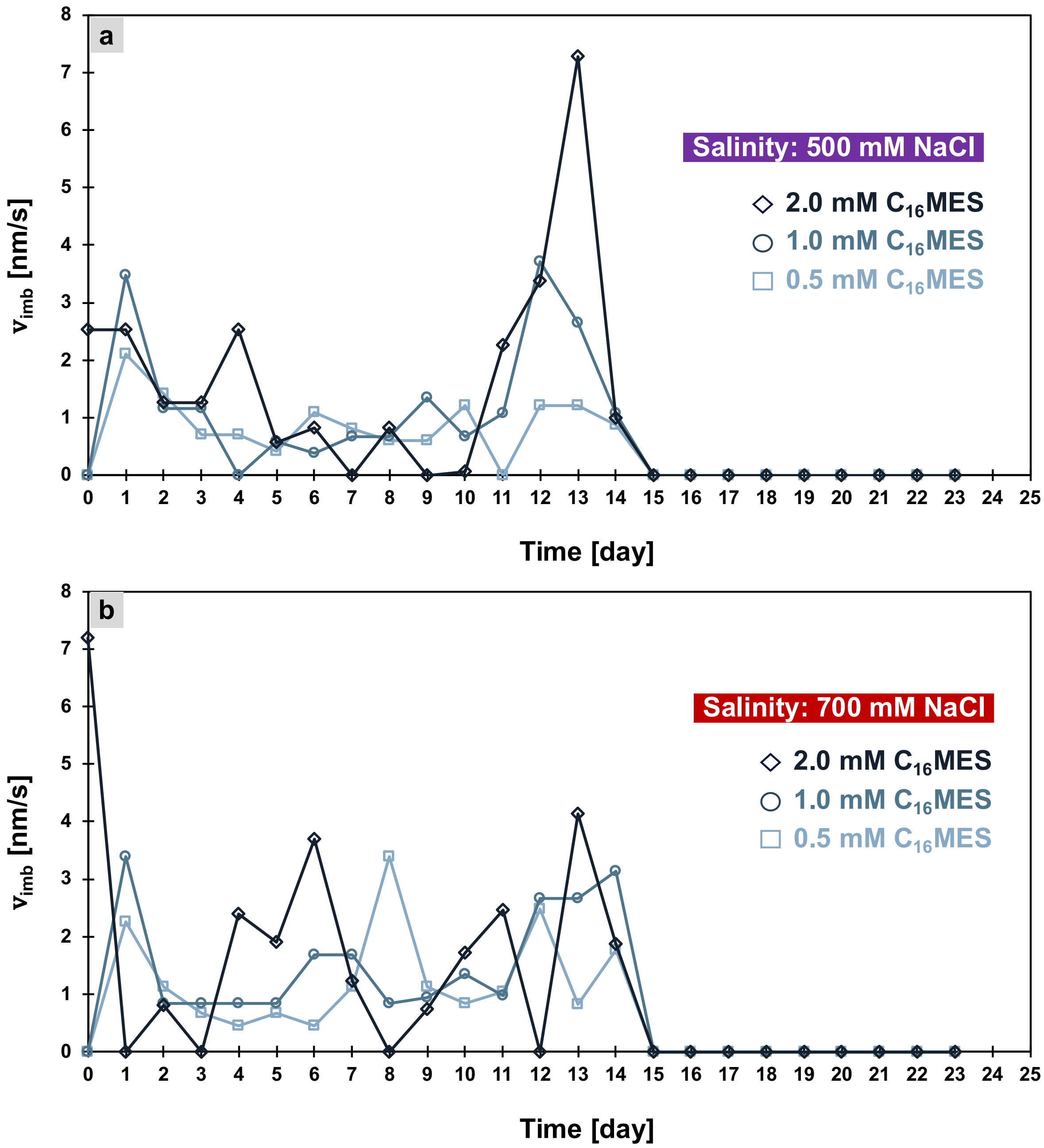

Figure S 3. Imbibition velocity $\mathrm{v_{imb}}$ profile from day 0 to 24. (a) Salinity at 500 mM NaCl and (b) 700 mM NaCl. Unit of velocity is nm/s or 10^{-9} m/s. Imbibition velocity is computed by $\mathrm{v_{imb}} = (dV/dt)/A$ where A is the cross-sectional area or rock cores.

REFERENCE

(1) Abdul-Majeed, G. H.; Abu Al-Soof, N. B. Estimation of Gas–Oil Surface Tension. *J Pet Sci Eng* 2000, *27* (3–4), 197–200. https://doi.org/10.1016/S0920-4105(00)00058-9.
